# Sociotechnical imaginaries of a secure future

**DOI:** 10.1186/s40309-021-00176-1

**Published:** 2021-06-21

**Authors:** Lars Gerhold, Edda Brandes

**Affiliations:** Working Group Interdisciplinary Security Research, Institute of Computer Science, Freie Universität Berlin, Carl-Heinrich-Becker-Weg 6-10, 12165 Berlin, Germany

**Keywords:** Security studies, Futures studies, Security foresight, Security technology, Scenario method, Public security, Sociotechnical development, Sociotechnical imaginaries, Technization of security

## Abstract

The article examines the increasingly important role played by technology in the domain of public security in Germany, illustrating its effects on social life. In order to illuminate developments that govern the adoption of security technologies and render them in their dependencies comprehensible, we present two plausible and consistent future scenarios for Germany 2035. Following Jasanoff and Kim, these scenarios are theoretically conceived as two competing “sociotechnical imaginaries” which implies different trajectories for shaping the future. In these imaginaries, security technologies condition social change, and vice versa, in a mutually interdependent process. On the basis of current literature in tandem with a structured scenario development process, we condensed the present sociotechnical imaginaries into two tangible future scenarios for the field of public security, illustrating its effects on how we live as a society. Our overarching goal is to identify key factors that will mediate future developments, and, by extension, to facilitate discussion on the type of future we find collectively desirable. The analysis of impact factors resulted in ten key factors that play a crucial role for the use of security technologies and serve as a leverage for shaping the future. Projections of these factors lead to two narrative scenarios “To Be Ahead” and “Turn Back The Clock”.

## Background

Technologies condition social change, yet social change also conditions technological development. In this way, technologies are inseparable from the broader social contexts in which they arise. And while the increasing deployment of security technology can to some extent assure greater security in an objective sense, “security” itself is a normative concept. Accordingly, our understanding of security is inextricably linked to values and beliefs that are constantly in flux. In the broader social discussion concerning the dangers we face and how they should be addressed (*viz*. “security culture”) [7], security technologies are one piece of the puzzle. For Jasanoff and Kim [25] and Stewart and Williams [53], security technologies and social developments mutually condition each other in a “co-evolutionary” process that precludes the identification of simple cause-and-effect relationships. Yet many security solutions—such as facial recognition technology and predictive policing—are not solely solutions to security-related problems. They are also an expression of innovation processes that are primarily driven by the private sector.

This paper presents the results of a research project titled STAGe (Security Technologies and their Impact on Social Life), which seeks to examine the increasingly prominent role played by technology in the domain of public security. Ever-greater responsibility for public security is being delegated to technological systems [37, 57]. From surveillance and identification systems to automated data analysis and storage, the influence of technological systems is now pervasive [5, 28]. Technological innovation has been proceeding at a breathtaking pace in recent years [5, 38], creating tensions with law governing police powers and individual privacy. Yet rapid technological advancements are not only changing how we live and communicate with each other as a society. They are also changing how we perceive the presence security technology in our everyday lives.

In addition to taking stock of current developments in the domain of security technology, the STAGe research project aims to illuminate how these developments impact public security and Germany society at large. In this paper, we sketch possible future developments in the area of security technology and consider in detail how they might reshape society.

Each hypothesized future is elaborated in a scenario development process that projects current developments into the future. In this connection, we strive to ensure that assumptions and their implications are internally consistent and plausible. One key benefit of these scenarios is that they can be used to illuminate how technological developments relate to ongoing political, social, and economic change. Whether or not a given hypothesized future will actually manifest is not the essential matter. Rather, the aim is to shed light on possible development paths in order to facilitate present-day decisions that will lay the groundwork for a desired future to occur.

The overarching research question that is addressed in this paper is as follows:

“How will security technologies look in Germany in 10 to 15 years, and what might this entail for public security and social life?”

In the next section of this paper, we couch this research question in a theoretical framework. We first introduce the premise that technology will place an increasingly prominent role in the domain of public security in coming years. Specifically, we describe our understanding of “technization” and “technology” in accordance with the current state of the literature. In line with the sociotechnical imaginaries of Jasanoff and Kim [25, 26], we view trends in security technology as the outcome of a co-evolutionary process. In this process, the most powerful competing vision of the future prevails. In order to represent the current future visions, we see the scenario methodology to be suitable to transform them into plausible and tangible depictions of the future. The third part then describes our empirical approach and the scenario development methodology, which are crucially informed by the domain of future research. In this section, the specific working steps of our study are presented in detail. In the fourth section, we present two hypothetical futures that emerge from our scenario process. These visions of future, which are based on the extrapolation of current developments, constitute two contrasting sociotechnical imaginaries.

## Literature review and theoretical considerations

### Technology and public security

The increasingly prominent role played by technology in the domain of public security has various causes. To some extent, it is the consequence of new technical possibilities in specific fields of application, e.g., in the fight against terrorism or crime [5]. However, processes of innovation in academia, industry, and government have also been propelling the adoption and economic success of security technologies [29, 45]. In this connection, the deployment of innovative security technologies have often allowed political actors to advertise that action is being taken to assure security—even when the tangible benefits of such action is questionable [58].

The terrorist attacks of September 11, 2001, can be seen as a watershed moment in the expanded use of technology to provide security. In addition to catalyzing the adoption of new security laws and guidelines, the attacks intensified the surveillance of monetary transactions, trade, and human movement. Following the September 11 attacks, a wide range of new security technologies were adopted [28, 29]. However, the September 11 attacks should be understood as intensifying already existent trends, as various events in prior decades, such as the IRA bombings of the 1970s, previously triggered the expanded use of surveillance technologies [5].

However, the expanded use of security technologies is not an inevitable consequence of such events. Rather, the decision to adopt a new technology or eliminate restrictions to its deployment is inherently bound up with a changing sociopolitical understanding of security and risk. In other words, the aforementioned historical incidents are intimately connected to shifting discourses around the issue of security [29]. To be sure, modern societies are becoming increasingly aware of their vulnerability to risk. In this connection, anxiety is triggered in particular by risks that are difficult to control given their complex and interconnected nature [3, 29]. As a result, ever more areas of social life are falling under the domain of security discourse [4]. During the twentieth century, security was elevated to the status of an important societal value [29]. Against this backdrop, the growing role of technology in security can be understood as motivated by the desire to establish control over risks. Security technologies not only promise to avert risks, by facilitating preventive measures, but also to render them transparent and manageable, by enabling monitoring and control [29].

Security technologies themselves are subject to constant change, which can be seen in the transition from reactive to proactive justifications for their deployment [54]. With the scope of a proactive deployment logic, the permanent and comprehensive adoption of security measures is viewed as essential to avert disruptive events. At the same time, today’s security technologies at not merely concrete objects, but also fluid expressions of power, as exemplified by the virtual nature of surveillance algorithms [2]. In this way, we find that surveillance technologies have evolved from *salient* to *silent technologies* [24], for they are invisible, inexpensive, and automated, while data gathering is ubiquitous and ongoing [34].

These developments are accompanied by numerous questions and challenges that have been widely discussed in the literature, but which can only be touched upon here [1, 9, 19, 39, 41, 44, 48]: Insofar as political decisions and the responsibility for establishing security are transferred to technical systems, questions are automatically raised as to their democratic legitimacy and amenability to control through the political process. Indeed, a lack of transparency in the provisioning of security is often associated with an “accountability deficit.” Algorithm-based decision making (ADM) implies action based on probability calculations rather than human judgment. When used to predict crimes, ADM can have discriminatory consequences—such as when specific neighborhoods are increasingly targeted, or when given individuals are subjected to inspection multiple times. In some cases, lack of accountability is the direct product of shallow deliberative processes that place hope in a “technological fix” to resolve complex social problems [10].

Against this backdrop, we view it as increasingly important to discuss and consider where the growth of security technologies may lead, so that we can arrive at proportional and consensually determined approaches for dealing with dangers and risks. Prior to engaging in such a discussion, however, is important to clarify what is meant by “technology” and “technization.”

### Technology as a concept

This project draws on current research in the field of sociotechnology, which views technology and society as a dualism, as technology conditions society, and vice versa. Technologies are “tools for artificially generating cause–effect relationships that can be used to produce certain desired outcomes with sufficient reliability and repeatability” ([50] p. 445^1^). In this way, technology enables a process that ushers in effects otherwise not possible or only achievable with greater effort [49]. As is customary in the sociology of technology, we select a narrow definition of technology that encompasses physical/material artifacts within the domain of security technologies [22].

Technology is invariably embedded in a sociotechnical constellation, whereby man and technology condition each other in a process of “co-constitution” [51]. The human being knows how to use a given technology, and it has been modified to suit human actors. Technology only produces its effects within the scope of this relationship [35, 51]. The adoption of a technology thus has societal implications. Technologies not only enable humans to perform actions beyond their natural capacities. They can shape human action to the point of coercion, and they are deeply integrated in everyday life, often to the degree of indispensability. In this way, they play a constitutive role in directing social and cultural change [22, 44, 46, 50]. However, technological advancement should not be viewed as the dominant driver of social change, as technology is itself a product of society. Accordingly, new technologies do not emerge solely from considerations regarding how to optimize existing practices. Rather, they are shaped by the values and ideas prevalent at a given socio-historical moment [43, 51]. The “technization of security” thus describes a process by which the security demands and expectations of society are increasingly fulfilled by technical systems. Prevailing norms and values are inherently inscribed into these systems [32], and their diffusion may depend on their social acceptance. Once implemented, security technologies develop a life of their own, and, in this way, may place constraints on the individual or on society [43].

Beyond explicitly desired effects, a technology may also generate unintended side effects [45]. Such side effects may be difficult to predict in today’s highly connected society. Furthermore, they may only be identifiable once the diffusion of a technology is well advanced [44]. However, once a technology is firmly embedded into society, it may be difficult to modify or replace it [6]. The foregoing observations have been repeatedly confirmed in recent decades. Accordingly, there is an increasing need to assess in advance the potential effects that may emerge from new technologies [33].

### Sociotechnical imaginaries

The societal and technical arrangements of the future that we hypothesize in relation to security technologies can be understood as “sociotechnical imaginaries,” a codified term in the literature that refers to “collectively held, institutionally stabilized, and publicly performed visions of desirable futures, animated by shared understandings of forms of social life and social order attainable through, and supportive of, advances in science and technology” ([26] p. 6). In this way, sociotechnical imaginaries are vehicles for the co-production and co-evolution of technology and society [26]. They are veritable images of the future, guiding activities toward a shared goal. In this connection, the actual capabilities and limitations of technical systems are less important than the vision of the future that is collectively held and desired. Aspects of the future imagined by individuals are taken up by a collective, invested with authority, and thereby manifested through collective action. Divergent imaginaries also stand in competition, each seeking to establish normative hegemony over an imaginable future. In the domain of public security, various normatively conditioned futures are possible. Indeed, there are a multitude of potential answers to the question of how the secure society of the future should look.

In the competition between imaginaries, some ultimately prevail, whether due to media-driven reinforcement [27, 47] or securitization processes, e.g., by being addressed intensively by politicians, as highly relevant for ensuring security [4]. In this connection, the concept of “security culture” can have a descriptive and explanatory effect, for it encompasses negotiative processes concerning what is to be considered a threat and how it is to be addressed [7]. In this way, security culture shapes normative imaginaries.

Sociotechnical imaginaries explicate normative ideas and have an action-guiding or animating character, since they facilitate the rendering of judgments about possible futures. In this way, the systematic description of sociotechnical imaginaries have the benefit of allowing us to recognize, understand, and describe future developments. At the same time, sociotechnical imaginaries disclose differences between different collectives. These differences are particularly evident with a view to the putative benefits and effectiveness of security technologies, which are often linked to divergent socio-cultural contexts. In this way, this approach offers the supplementary advantage of allowing us to reflect on the socio-cultural contexts that form collective visions of desirable and undesirable futures.

## Methods

In this study, we rely on the scenario technique, which allows us to present sociotechnical imaginaries in form of plausible and consistent depictions of the future. The competing sociotechnical imaginaries can thus be condensed into descriptive and tangible scenarios. We aim to show which imaginaries are currently dominating the debate and competing with each other by developing futures based on these imaginaries. By pointing possible futures out and putting them up for discussion, we also put their origin, i.e., the current imaginaries, up for discussion. Technical innovations that are under development or envisioned for the future represent the primary building blocks of our scenarios. We understand scenarios as “logical, plausible and realistic situations based on today’s knowledge, therefore giving an approximate idea of how the future may look. Whether, one day, the portrayed “possible future” does occur, is always uncertain” [[18], p.71]. However, we do not take specific probability of their occurrence into account. Rather, after conducting a comprehensive review of the literature, we identify key factors relevant to the future of public security and place them in relation to each other. The characteristics of these factors are varied and then ascribed to possible future developments. Each resulting set of factors is assessed with a view to plausibility and consistency. These factor sets each yield a competing sociotechnical imaginary.

Our methodological approach is grounded in the notion that the future can be actively shaped. While we must acknowledge that the future is not fully predictable and controllable, it is also not wholly random or chaotic [15, 31]. Indeed, the discipline of future studies holds that a number of divergent and alternative futures are possible [15, 20]. Furthermore, our current actions and decisions help determine which of them will prevail [31]. The goal of our methodological approach is to present tangible, plausible, and consistent images of the future, which allows the reflection of current debates about security technologies and can inform which actions in the present are best-suited to bringing about desired future states [21, 31, 40].

**Fig. 1:**
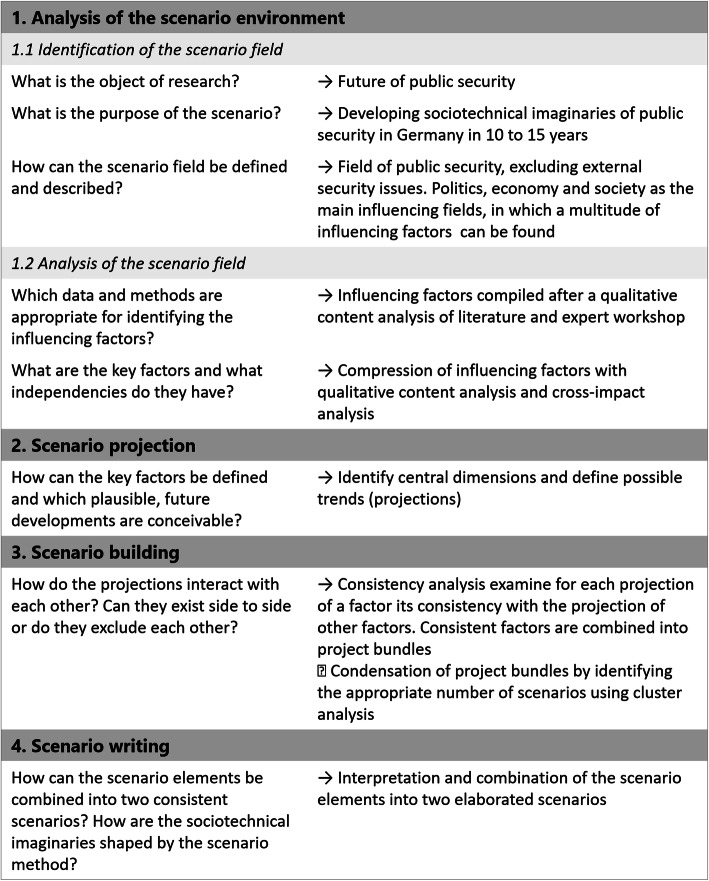
The four steps of scenario development

In the following, we describe our methods for the development of each scenario in greater detail. Our work was composed of four working steps (see Fig. 1), which is a typical subdivision in the literature on the scenario method [15, 23].

### Analysis of the scenario environment

This first step aims to delimit and more closely define the subject of research by setting the focus, the time frame, and the geographical scope [42]. Our broader scenario environment is “public security in Germany” and our specific subject of research is “security technologies.” We have selected 10 to 15 years as a chronological horizon for our analysis. This timeframe seems suitable, for it is long enough to consider the effects resulting from the broad adoption of technologies currently at a nascent stage, yet not so long so as necessitate speculation concerning technological advancements that are marked by significant uncertainty.

In this step, we also analyze the factors influencing our subject of research, and to what extent. We first performed an explorative horizon scanning process, which is an increasingly used technique in the scenario methodology. It has the advantage of less bias in comparison to conventional forecasting methods [56]. This work considered a range of sources, from academic papers and news reports to podcasts and policy papers, while considering the following questions: “Which security technologies are already in use or under development worldwide? What impacts will result from the implementation of these new technologies?” We identified sources relevant to these questions while considering two criteria: First, we only considered sources that addressed one or more technologies in connection with security as well as society, politics, or economics. Second, we only selected sources that discussed technologies in use or currently under development. This approach yielded collection of 77 media stories and 32 policy papers published between 2013 and 2019. The media stories originated from various national and international periodicals and news sites. We also gathered position papers released by German political parties as well as policy papers published by political institutions and research organizations in Germany, the US, and UK. Papers were taken into consideration insofar as they contained policy recommendations or assessments related to security technologies. Finally, a workshop with experts from diverse professional backgrounds was held to identify current developments in public security in Germany.

**Fig. 2:**
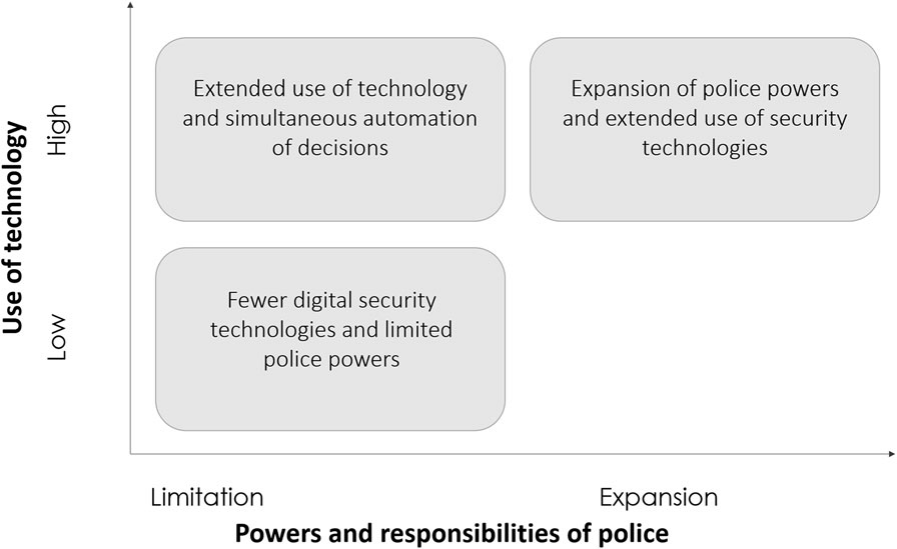
Influence matrix

Based on these data, we identified various factors that would appear to exert an influence over the development of public security in coming years. They comprise parameters, developments, and trends [31]. To this end, we conducted a qualitative assessment of the source material while identifying relevant factors in various domains, including politics, economics, technology, law, and the environment. As part of a multi-stage collaborative process designed to augment the validity of our assessments, we distilled the factors thus identified into a smaller list of 15 impact factors. We then considered these impact factors in relation to each other with the aim to identify key drivers of change while also revealing interdependencies between factors. In the scope of an impact assessment, three researchers individually valuated for each impact factor the respective influence on another factor on an ordinal scale. For each factor, we sum up how much the researchers estimate the influence of one factor to another (*active sum*). We also sum up how much one factor was influenced by another (*passive sum*). Subsequently, the three evaluations were compared. Although the researchers had different views on how high the influence of a certain factor is, no strongly deviating evaluations were found. In the next step, we calculated mean values from the individual evaluations, which are presented in Fig. 2.

The active and passive sums have been divided into four quadrants according to the mean values on each respective axis. Our interpretation of the quadrants is based on Kosow and Gaßner [31] and von Reibnitz [55]. Quadrant I contains dynamic key factors. They are characterized by a high degree of ambivalence and have strong reciprocal relationships with other factors. They are also particularly relevant for the design of strategies for the future. Quadrant II contains driving factors. These active or impulsive factors operate as a “lever” for future developments. Quadrant III contains inert factors that are not amenable to influence and also have little impact. The final Quadrant IV contains reactive factors. These factors are strongly driven by other factors and can therefore serve as a bellwether for other developments. We consider the factors in Quadrants I, II, and IV to be key factors. Figure 2 presents our results. The figure contains ten key factors that will drive technology’s increasing influence over security in Germany up to 2035. It also contains five inert factors that we deem less relevant.

### Scenario projections

Based on our data from the literature review, we developed projections for each key factor along two different lines of growth. We identified the dimensions collaboratively in a dedicated review of our qualitative literature analysis by considering on which dimensions all coding would fit. These dimensions seek to anticipate the development of each factor, as explained below. This is a necessary step for our subsequent consistency analysis, in order to ensure each key factor remains internally coherent.

**Fig. 3:**
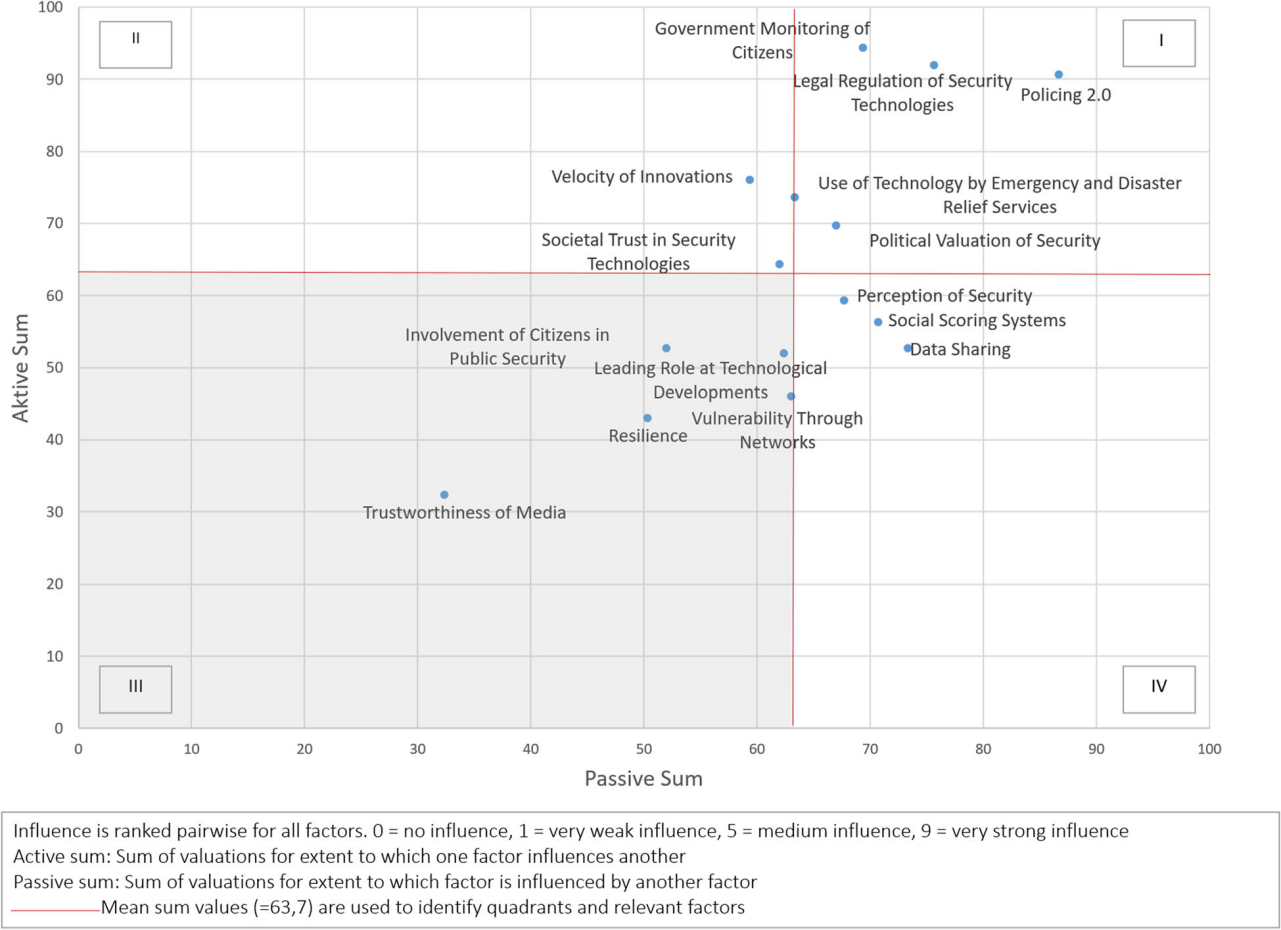
Example projections for key factor “Policing 2.0”

In the following, we demonstrate this step with reference to one key factor, “Policing 2.0”: A significant portion of the codings relate to a number of security technologies that could potentially be used in the domain of policing^2^. Derived from this, security technologies will significantly influence the future of policing, so the extent to which such technologies are actually employed by the police was selected as the first dimension (see Fig. 3, Y-Scale). Our analysis additionally revealed that the scope of the powers and duties accorded to the police will represent an important aspect of policing in the coming years. Specifically, security technologies are already transforming how police do their work, thus creating frictions with existing rules governing police powers^3^. Accordingly, in our graph, the X-axis maps the scope of police authority, which can be restricted or expanded in the future. From these two dimensions, three plausible projections emerge (see Fig. 3) for we rejected as implausible a future in which new technology is not used while police powers are simultaneously increased.

A list of the projections for each key factor can be found in Table 1.

### Scenario building

The software application Scenario Manager (SCMI) was used to create the scenarios. First, the projections were placed into relation to each other in a consistency analysis. Specifically, after the projected key factors were compared in pairs, we decided consensually whether both projections could occur simultaneously, without contradiction. Using a consistency matrix, pairwise consistency ratings were assigned using a scale of 1 (total inconsistency) to 5 (strong mutual support). For example, greater reliance on technology by the police was seen as very consistent with a high rate of innovation in the future. By contrast, we considered a slow pace of innovation in combination with the adoption of new technologies by government authorities responsible for security to be two developments unlikely to occur simultaneously in the future.

To minimize the influence of personal bias, individual assessments were first made by each researcher and then, in the case of divergent assessments, an agreement was reached by consensus. This consistency analysis resulted in a large number of “projection bundles.” These bundles represent internally consistent pictures of the future. In a subsequent step, these projection bundles were tested in for similarities. Therefore, a cluster analysis was performed to compress similar projection bundles to be consistent to a manageable number of future [11]. The cluster analysis and resulting scree diagram indicated that the projection bundles can be most reasonably divided into two scenarios. Accordingly, we opted for two distinctive rather than three or more less delimitable scenarios. This offers the advantage that projections are clearly assigned to a factor, which guides the scenario writing more clearly. In addition, the differences between the scenarios are easier to grasp. Table 1 shows, for each scenario, the share attributable to each projection within the respective projection bundle. Percentages above 65^4^ indicate a clear projection within the scenario. If alternative projections are also conceivable, but the percentage is less than 25, these should not be considered in scenario-writing stage. A projection is considered characteristic of a scenario if it only appears in that scenario. The unique, characteristic and alternative projections are the “scenario elements” of each the scenario [11]. In the scenario-writing stage, the insights from the literature are used to interpret these scenario elements and elaborate two sociotechnical imaginaries.

### Scenario writing

The scenario-writing stage aims to translate the scenario elements that have been distilled in a systematic way into consistent, plausible, and easily accessible pictures of future [17, 52]. In the following, we use a written format that addresses the key factors step-by-step, differentiating them into a coherent narrative about a possible future. To this end, we assemble the insights gathered thus far and seek to synthesize them into a seamless and consistent picture [52]. In each scenario, the individual key factors are contextualized and further developed. Potential manifestations of the factors are elaborated and used to flesh out the scenarios, based on the insights developed in the literature review. We differentiate between driving (active), driven (reactive), and dynamic key factors. In our scenario writing, we strive for a factual presentation, without literary embellishments. Nevertheless, our scenario writing is based in part on a creative process that is influenced by the experiences and assessments of the authors. Accordingly, scenario writing can never be fully objective or devoid of individual bias. Subjective evaluations were reduced based on critical dialog between the authors. The scenarios are thus grounded in the findings of the preceding methodological steps (see Table 1). The two scenarios are compared to each other based on the key factors. Driving, driven, and dynamic key factors (Table 1) are emphasized according to their role in each narrative. Inert factors are displayed as well, but nor projections were made.

## Results

### Factors, projections, and relevance for the scenarios

Table 1 shows our results for the projections of the key factors, as well as their relevance for the scenarios.

**Table 1:** Key factors

Title	Description	Projections	Scenario 1: Policing 2.0	Scenario 2: Turning Back the Clock
Abbreviated title for the factor Active sum Passive sum Mean=63.7	Explanation of the factors	Possible developments for key factors	Relevance of the projection in the scenario (only percentages above the 25% threshold are listed, as recommended by the SCMI manual)	Relevance of projection in scenario (only percentages above the 25% threshold are listed, as recommended by the SCMI manual)
*Driving factors*				
Velocity of Innovation Active sum: 76.0 Passive sum: 59.3	The velocity of innovation describes the ability of a society (including the government) to develop, modify, and integrate new security technologies. Various catalysts – such as openness to innovation and funding support – encourage the development and adoption of new technologies, and, more generally, the use of technologies to address security problems.	High velocity of innovation, low level of regulation, high diffusion of technology solutions, risks due to rapid and unproven use	–	100
		Low velocity of innovation due to regulation, skepticism on the part of consumers, but also prudence and rigor when developing technologies	100	–
Use of Technology by Emergency and Disaster Relief Services Active sum: 73.6 Passive sum: 63.3	Emergency and Disaster Relief Services rely on new technologies. For example, drones are generating situation pictures; exoskeletons are worn by firefighters; VR technology is used for training purposes; and AI and drones are used to evacuate large public gatherings in emergencies.	Large-scale deployment of new security technologies	67.6	-
		Rejection of new security technologies		50
		Combination of new and conventional security technologies	32.4	-
		Unsuccessful adoption of new security technologies by Emergency and Disaster Relief Services	-	50
Societal Trust in Security Technologies Active sum: 64.3 Passive sum: 62.0	The factor describes the relationship between social trust in security technologies and their use. Transparency regarding the motivations for deploying security technologies, including possible implications, increases social trust. On the basis of such transparency, individuals can develop a perspective on how a given technology works and its suitability for meeting defined aims, and can thus decide whether they favor or oppose adoption.	Use of technology despite lack of social trust	18.9	–
		Limited use of technology due to societal misgivings	–	100
		No societal misgivings related to use of technology	81.1	–
*Driven factors*				
Perception of Security Active sum: 59.3 Passive sum: 67.7	The population’s perception of security is subjective and is determined by individually selected and weighted criteria. It has a cognitive dimension (perceived likelihood of danger and threat) and an affective dimension (fear, worry). There is often a pronounced discrepancy between subjective perceptions and objective threats to public security.	People perceive security appropriately (correspondence between subjective perception and statistical security situation).	9.5	27.3
		People feel more insecure than is statistically appropriate.	78.4	–
		People feel overly secure and do not perceive statistically significant risks.	12.2	72.7
Social Scoring Systems Active sum: 56.3 Passive sum: 70.7	Social scoring is becoming increasingly common around the world. Data on individual behavior are increasingly evaluated numerically, as such assessments are supposedly objective. While the composition of a social score is generally not transparent, its impacts for an individual or firm can be significant. In some countries, access to services and goods depends on one’s social score.	Social scoring systems (many of them intransparent) are used by various actors	20.6	–
		Social scoring is only allowed if it is transparent; individuals recover their control over personal data.	–	100
		Scoring is massively restricted and must be completely transparent. People can independently check what data has been collected and processed	79.4	-
Data Sharing Active sum: 52.7 Passive sum: 73.3	The exchange and sharing of data may be voluntary or forced, legal or illegal, and may take place between individuals, governments, and firms. This key factor highlights the importance of the data that are generated and exchanged by security technologies.	States collect and share the personal data of citizens on a legal basis	64.7	-
		Individuals voluntarily share data in exchange for goods and services	35.5	50
		- Data are collected and shared without legal basis	-	-
		Data are shared voluntary, but then used illegally	–	50
*Dynamic factors*				
Government Monitoring of Citizens Active sum: 94.3 Passive sum: 69.3	This factor describes government surveillance as a possible driver of the technization of security. The increasing prevalence of surveillance and detection systems, combined with improved data storage and analysis capabilities, enable the comprehensive surveillance of the population, in both the public and private domains. The expansion is accompanied by a growing political dialog regarding the compatibility of surveillance with democratic values.	Digital forms of surveillance are on the rise because they are more efficient than conventional techniques	100	–
		Digital forms of surveillance decline, as they are vulnerable to cyberattacks	–	–
		Surveillance as a whole becomes less prevalent due to social opposition	–	100
Legal Regulation of Security Technologies Active sum: 92 Passive sum: 75.7	Standards, laws, and regulations set forth legal arrangements for the use of technologies.	The use and deployment of security technologies is regulated internationally	14.7	66.7
		The use and deployment of security technologies is regulated nationally	85.3	33.3
		There is little or no regulation of the use and deployment of security technologies	–	–
Policing 2.0 Active sum: 90.7 Passive sum: 75.7	Technological innovations lead to new forms of police work. For example, the police use genetic databases to convict criminals; predictive policing is used by many police departments; criminals can now be taken into preventive custody; body cams are a standard piece of equipment; government malware is used to track down criminals and terrorists; and AI predicts convict recidivism rates. These new technologies are linked to new powers, rights and duties for police officers.	Broadened use of technology, leading to automation instead of assistance	67.6	–
		Fewer digital security technologies; curtailment of police powers	–	100
		Expansion of police powers and broader use of new security technologies	32.4	–
Political Valuation of Security Active sum: 69.7 Passive sum: 67.0	Determining the proper balance between freedom and security is one aim of political discourse. In this discourse, emphasis is placed on the extent to which the granting of freedoms is possible and/or desirable in the context of ensuring security, and vice versa.	Policymakers prioritize security over freedom, driving the expansion of security technologies	73.5	–
		Policymakers prioritize freedom over security, curtailing the expansion of security technologies	–	100
		The government can provide security by expanding the use security technologies. However, these are regulated to ensure they do not restrict the freedoms of the citizenry	26.5	-
*Inert factors (no projections are made)*				
Vulnerability through Networks Active sum: 46.0 Passive sum: 63.0	The networking of IT systems and physical objects engender new vulnerabilities, not only for security systems themselves, but also for their users.	-	-	-
Resilience Active sum: 4.0 Passive sum: 50.3	Resilience describes the ability of systems to withstand, prepare for, recover from and adapt to events or processes with negative consequences, whether human, technological, or environmental in nature.	-	-	-
Involvement of Citizens in Public Security Active sum: 52.7 Passive sum: 52.0	The digital transformation offers new opportunities for public authorities to inform or involve the citizenry in their work. The question remains to what extent the public should be involved in planning for emergency situations, in order to ensure preparedness.	-	-	-
Leading Role in Technological Developments Active sum: 52.0 Passive sum: 62.3	This key factor focuses on firms and public authorities that (1) produce, support, or develop security technologies, or (2) significantly influence technological developments with their funding, ideas, or decisions. These actors thus have a directive role with regard to security technologies, and can thus enforce their values, standards, and guidelines.	-	-	-
Trustworthiness of Media Active sum: 32.3 Passive sum: 32.3	The fracturing of the media landscape creates a situation in which people believe less and less what they read or hear in the mainstream media. In addition, “filter bubbles” reinforce individuals’ existing opinions and perspectives.	-	-	-

### The scenarios

**Table:** 

Narrative Scenario 1: “To Be Ahead”	Key Factor	Narrative Scenario 2: “Turning Back the Clock”
The year is 2035, and in Germany public security is now considered an inalienable right. Almost without exception, public security is understood as the absence of threat to life or limb, especially in the public realm. Germany has been repeatedly struck by terrorist attacks – from knifings on the street to cyberattacks. The prevailing political credo is that “everything that can be done to protect the population will be undertaken.” Although policymakers have passed laws to prevent the arbitrary use of security measures and to protect against a surveillance state, there are significant loopholes that enterprising German firms are able to exploit. Government subsidies for the development of security technologies have strengthened the German economy.	*Political Valuation of Security*	The following incident makes headlines in 2022: Despite having done nothing wrong, a young man visiting a music festival is arrested and spends several weeks in prison. While he was at the festival, facial recognition software flagged him as an imminent threat. In accordance with powers granted to the police, he was thus taken into “preventive custody.” An independent scientific institute reviewed the incident and determined that an error had been made. However, all involved parties disavow responsibility. Attitudes toward mass surveillance in Germany underwent a cultural shift after what happened in 2022. The surveillance of public space went from a fringe concern to a major political issue, and the majority of citizens suddenly felt that mass surveillance placed them under “general suspicion” in a manner that was not warranted. A consensus arose that security technologies are not infallible. Accordingly, by 2035, society at large is opposed to mass surveillance, and associated preventive measures. There is a consensus that security should be addressed by other means – such as improved resources for the police – that is, measures that do not restrict individual freedom.
Regulations and associated legal restrictions have been kept to a minimum, thus supporting more rapid innovation. Although there are risks posed by the rapid and untested deployment of new technologies, successes quickly emerge, which in turn spur a willingness to innovate. Security technology “made in Germany” becomes a major source of export receipts. Regulators are unable to keep up with the rapid diffusion of technological innovation. Adequate regulatory responses have thus become difficult, but public calls for action have also diminished. Public outcry is limited because new technologies are closely adapted to people’s everyday needs, and thus meet with approval. Citizens record crimes with their smartphones and send notifications directly to the police. When citizens record a crime with a specific emergency app, the police are automatically dispatched, and the video recording provides the police with advance information about the incident. Warning apps provide accurate information about impending severe weather, forest fires, industrial accidents, utility outages, and more, and also suggest specific courses of action. These apps thus ensure an improved societal response to emergencies. One innovation – implemented during the 2020 Corona pandemic – is the measurement of air quality in restaurants and stores. Air filtration systems can now measure the virus load in the air, and communicate such information to potential visitors. When defined thresholds are exceeded, the store or restaurant is forced to shut down temporarily. Technologies such as facial recognition and fingerprint scans are now standard at the airport. People no longer carry identification documents, which are considered unreliable. At a police or border checkpoint, and at the hospital, individuals verify their identify with a fingerprint scan. Overall, there is general acceptance among experts and the broader public that security technologies contribute effectively to addressing security problems.	*Velocity of Innovation*	A libertarian current in the political debate is responsible for a critical stance on new technologies. Security technologies are no longer seen as a panacea that can provide answers to sociopolitical problems. The risk of unintended side effects and ethical concerns also play a prominent role in the political debate. As a result, a number of regulatory requirements have been adopted. One key requirement is that such technologies must be assessed intensively by independent evaluators prior to large-scale use. The costly process leads to a slowdown in the velocity of innovation. In 2035, comprehensive impact assessments must be performed when new security technologies are planned, developed, or deployed. The slow pace of innovation in the area of security technology ensures that other areas of society have an opportunity to “keep up.” In particular, there is sufficient time for appropriate regulations to be adopted; for security weaknesses to be addressed; and for education and training regarding technologies’ proper use. This has made it very costly for German companies to introduce new security technologies to the market. However, when such technologies are introduced, they have an international reputation for fulfilling high ethical and quality standards. Products made in Germany are known for being innovative, user friendly, and secure – particularly when it comes to personal data. By contrast, other countries with fewer regulatory restrictions are generating larger economic gains by rolling out more innovative security technologies. As a result, German technology companies are increasingly conducting their R&D activities in other countries, and also tend to look abroad for sales opportunities.
Surveillance is a key area of technological innovation. Governments around the globe rely on surveillance to ensure public security, especially when it comes to preventing terrorist attacks. The German government has also expanded surveillance. “If you have nothing to hide, you have nothing to fear” is the watchword of the day; in the eyes of the government, only those who commit crimes have a reason to fear repercussions. Security technologies such as facial recognition are seen as much more effective and accurate than conventional means of policing when it comes to identifying suspects and monitoring public space. Illegal immigration is prevented by the creation of European databases, universal smartphone access, and new security technologies. Large-scale surveillance technologies make it impossible to “go underground” and are accepted as an “invisible overseer” of public order.	*Government Monitoring of Citizens*	In 2035, the German government is increasingly concerned about the security economy focusing on sales markets abroad, in part because of the rapid rise of surveillance in other countries, such as the US and China. Policymakers conclude that more surveillance does not translate into greater security. Accordingly, policymakers repeatedly point to the culture of suspicion that comes with increasing surveillance. In 2035, skepticism of surveillance remains high among the populace, and government sensitivity to this issue gives the electorate the feeling that they are being heard. In the early 2020s, numerous large-scale demonstrations had taken place in opposition to the surveillance state. Campaigns for the right to the anonymity were launched under the slogan “My Face, My Right”
In 2035, the police now rely extensively on technological solutions when combating all forms of crime. In the past 10 to 15 years, a slow response on the part of legislators in combination with rapid innovation has enabled a dramatic transformation in police work. In the area of burglary and violent crime, experience shows that surveillance technologies are much more efficient and effective than police patrols. While new technologies have failed to effectively deter crimes, apprehension rates have significantly improved. Cities with over 100,000 inhabitants now rely on several technologies in unison: Cameras with sophisticated sensor technology enable precise facial recognition, even when individuals are wearing patterned clothing or conspicuous glasses. Missing persons are essentially a thing of the past, as the intelligent cameras can locate anyone. The surveillance systems can even detect people based on their gait and other movement patterns. Suspicious activities are also monitored: Conspicuous behaviors that are considered precursors of violent acts or theft are identified and immediately investigated. Such crime detection systems are now extremely reliable; errors only occur in rare instances. Surveillance systems also sift through suspect databases on an ongoing basis. Flagged individuals are quickly arrested by police offers, following verification of their identity. Police spokespersons emphasize that human verification is always performed prior to an arrest. In this way, the automated identification of a suspect is reviewed and confirmed by a human police officer. This procedure aims to augment public acceptance of the surveillance systems. However, the critics note that the technical systems are better than their human counterparts. In this way, predictive policing is now major aspect of police work. “Always a step ahead” is the watchword of the day. The police seek to prevent criminal acts *before* they occur. After much deliberation, predictive policing tools were adopted at the state and federal levels in the mid-2020s, paving the way for the standardization and integration of various systems. Now, in 2035, predictions are not only made on the basis of recorded crimes, but also take into account local demographics, urban structures, income statistics, and socio-cultural characteristics. Furthermore, as an increasing number of crimes are committed with the aid of internet-based communications, legislative changes have empowered the police to access the internet records of suspects, even in the case of minor offenses. Electronic communications are scanned for relevant keyword combinations; this allows individuals planning criminal activities to be identified in advance and placed under preventive arrest. Criminals on parole are tracked with electronic ankle bracelets. These location data flow into predictive policing systems. Individuals with a high predicted likelihood of committing a crime are subjected to particularly comprehensive surveillance – and they are also made aware of this fact.	*Policing 2.0*	Political developments have repercussions for everyday police work. The police are once against “on the beat”. Police officers seek to be approachable while developing ties to the local community, thus reinvigorating the traditional model of policing. This form of communal police work is made possible by hiring more officers, increasing pay, and investing in better equipment. While technological solutions are used by the police, their primary purpose is to augment the efficiency of internal processes and communication with the public. All officers are equipped with smartphones and tablets, but these devices have a restricted range of functionality, and are centrally administered. Body cams have been phased out, but it is possible in certain cases to unlock the camera on an officer’s mobile device. Top priority is assigned to ensuring the security of IT infrastructure used by the police. Breaches of IT security in years prior to 2035 have made this a particularly sensitive issue. Personal data gathered by the police are kept strictly confidential.
Fire departments, paramedics, and disaster relief organizations also rely on new technologies. While such emergency services were slow to adopt new technologies in the 2020s, they now employ them on a wide-scale. Drones play a particularly important role, for they allow emergency services to safely reconnoiter large-scale accidents and natural catastrophes, such as areas impacted by flooding. In addition to providing a live video feed of the disaster area, the drone can automatically identify objects of relevance, including fire hydrants, gas stations, and potential casualties. Such details can be automatically fed to augmented reality goggles worn by emergency service workers on the scene. At the same time, emergency workers can see details concerning ambient temperature and the composition of the atmosphere, thanks to sensors in their clothing. Following the failures of the 2020s, Germany’s disaster response systems were revamped and enhanced with new capabilities. Every citizen can now be warned effectively. Furthermore, the individual systems are effectively integrated, and feature a redundant, extremely reliable design. Disaster events simultaneously trigger sirens, SMS notifications, and social media alerts. Radio and television broadcasts are also interrupted with emergency notifications. The communication of emergency information through social media is now a firmly established practice among emergency and disaster relief services. Each emergency service organization has its own social media department, and these departments can be quite sizable. Big data analytics are used to assess the social media environment and develop a communications strategy. AI-based software systems respond automatically to common questions and factually incorrect posts. They also issue “rumor control notifications” if falsehoods begin to spread.	*Use of Technology by Emergency and Disaster Relief Services*	While the police have adopted numerous innovations by 2035, other emergency services – such as the fire department, paramedics and disaster relief agencies – largely rely on traditional technologies. This is partially due to reliability problems: drones with autopilot functionality have repeatedly crashed, or have delivered erroneous information. In one case, an emergency worker was even killed by an errant drone. Accordingly, drones are only used in rare cases, and only when operated by a trained pilot. Augmented reality solutions have also failed to catch on. “AR clouds rather than clarifies,” is a popular refrain. There are just a few virtual training centers in Germany. They are operated by large organizations, and are used to practice responses to rare events, such as terrorist attacks or nuclear accidents. Following a series of failures by the country’s disaster warning systems, policymakers ultimately opted for a hybrid solution: In addition to sirens, traditional media – television, radio – as well as major social media channels are used to issue warnings to the population.
By 2030, most people are in favor of placing advanced security technologies in the hands of the police and emergency services, because the results speak for themselves. Burglary statistics have fallen significantly, and there has also been a decrease in assaults. When disasters do occur, effective warnings are issued, and emergency personnel have achieved considerable successes in rescuing endangered or injured individuals. In addition, the police and disaster relief officials have made significant efforts to communicate with the broader public. The citizenry can visit dedicated websites to learn more about the technological systems being used, and officials answer questions immediately. Furthermore, answers to common questions are openly publicized. The police and emergency services maintain special blogs to document their operations, and independent evaluations are published openly to everyone. Effective oversight mechanisms augment public consent for security technologies. Body cam footage, for example, is regularly placed online for public inspection. Police officers, firefighters and paramedics are also regularly monitored to prevent possible misconduct. While some police technologies have been controversial – such as crime maps, which provide detailed information about criminal activity on a neighborhood basis – this has not prevented their implementation. As a general rule, once a technology has been successfully implemented, it remains in place.	*Societal Trust in Security Technologies*	By 2035, the citizenry is quite skeptical of security technologies, not least due to the high profile incidents of past years. “If you have nothing to hide, you have nothing to fear” is an adage that no longer has currency. Furthermore, disaster warning apps are no longer in use. The police have been particularly hard hit by negative press. Online surveillance by the police aroused public outrage after several incidents came to light in which police officials were found spying on relatives and former lovers. A few months later, bank accounts across Germany were emptied in a wave of hacker attacks. It was uncovered that the attacks were made possible by a weakness known to the police, which was exploited to monitor financial transactions. Public outcry resulted in massive restrictions to online surveillance by the police. New limits were also placed on the ability of the police to access personal data gathered by telecommunications companies. Subsequently, in 2028, following a hack of police databases, the addresses of prominent individuals were leaked to the public. As a result, many of them received threatening letters, or had their houses vandalized. In the media circus that ensued, numerous celebrities and politicians stepped forward to admit they had been victimized. In 2026, evidence also emerged that sensitive police data had been sold by a rogue official. This allowed criminal gangs to identify lucrative targets for burglary. At the same time, they were able to manipulate predictive software in order to foil their apprehension. As an outcome of these events, by 2035 the public has little confidence in the use of security technologies by the police. The public insists on a traditional approach to policing with human officers, rather than a reliance on digital technologies.
Ultimately, public support for surveillance is a reflection of the fact that people still feel uncertain in the public realm, despite new technologies. Particularly among women and the elderly, public spaces are not considered particularly safe. Among society at large, there is also a significant fear of terrorism, which is further augmented by each terror incident. Surveys show that a significant percentage of the populace feel unsafe when walking at night, when attending large public events, or when taking public transportation. Accordingly, they welcome the fact that intelligent camera systems monitor large-scale events, and that buses and trains have emergency call buttons. A lingering sense of uncertainty among the populace is by no means a novel phenomenon. Security concerns have been persistent over many years, despite various informational campaigns designed to place threats to public security in proper perspective. A vicious circle has taken hold in which isolated events are instrumentalized by political actors and reported widely in the media. This aggravates fears among the citizenry, while also augmenting a willingness to accept new security technologies.	*Perceptions of Security*	In 2035, life in Germany is more secure than ever before. Overall life expectancy continues to rise, and health care is of top quality. Once the Coronavirus pandemic of 2020/21 was defeated, business and leisure travel experienced a dramatic resurgence. Now, in 2035, freedom of movement remains a highly valued good. Yet people realize that freedom always entails some element of risk. The ability to travel the world while enjoying unrestricted access to advanced telecommunication technologies – there is a general understanding that these opportunities are not without their perils.
In the 2020s, public concerns about security gave rise to numerous legal changes. The powers enjoyed by the police were expanded and a legal foundation was created for the use of advanced security technologies by emergency services and disaster relief authorities. The sense of uncertainty among the populace was particularly high following the terrorist attacks of the 2020s. Each new incident led to the further expansion of police powers, and ever more invasive security technologies were adopted as the decade wore on. Public opinion increasingly favored a proactive rather than reactive stance to the policing of terrorist threats; this, in turn, shaped the legal basis for the deployment of new security technologies. Constant and far-reaching surveillance was viewed as justified to avert terrorist attacks, given the scale of the damage they inflicted. In 2035, Germany’s legal framework for security technologies entails limitations to data privacy and other rights. However, the law also enshrines ethical standards for the responsible use of information gathered in surveillance operations.	*Legal Regulation of Security Technologies*	Following the hasty implementation of new security technologies in the early 2020s – with attendant negative consequences – an international treaty was signed to regulate the ethical use of artificial intelligence. The primary goal of this treaty was to protect data privacy and prevent international espionage. In 2030, European legislators initiated multi-year deliberations on AI ethics, which culminated in the adoption of mandatory standards for all EU states. Among other things, these standards call for the use of “unbiased” data when developing AI systems, in order to prevent discrimination. Surveillance technologies must fulfill numerous criteria before they can be implemented in Germany. Other EU countries are entrusted with auditing such systems before they are implemented. The legal framework for surveillance technologies contains broad definitional categories, such that legal changes are not required every time a new solution is developed by industry. In this way, the legal environment prevents the hasty introduction of surveillance measures as a means of averting terrorist attacks. Before a surveillance technology is used, regulators consider whether it is compatible with individual rights, and whether it can make a tangible contribution to foiling criminal acts or prosecuting lawbreakers. Internationally, the EU plays a vanguard role in the regulation of new security technology; its progressive approach to regulation is seen as crucial to preventing the rise of a surveillance state.
Laws introduced in recent years have made it possible to store data about Germany’s citizens on a grand scale. Using these data, the effectiveness of facial recognition software and predictive policing has been significantly improved. At the same time, in the event of a major emergencies and mass casualty events, it is now much easier to identify victims and inform relatives. Public authorities are very transparent about the data they possess and why they are needed. Among the citizenry, consent for data collection practices is therefore high. Citizens are generally willingly to allow government agencies to take their fingerprints, conduct retinal scans, or collect other personal or biometric data. Laws have also been passed that allow data collected by companies – such as airlines or search engine providers – to be shared with the government for law enforcement purposes. For large sections of the population, the government’s judicious handling of personal data legitimizes data collection practices, as confirmed by public opinion surveys and outreach initiatives. One regularly touted example is the case of Xavier J., who had ordered large quantities of bomb-making material online. He had also searched for bombing-making instructions, was active in anti-government chat rooms, and had booked flights to Cuba. These suspicious activities triggered further investigation by the police. Following several days of surveillance and a search of Xavier J.’s domicile, he was arrested along with several members of his criminal network.	*Data Sharing*	At the national level, there was little regulation of new policing measures and the security technologies tied to them in the early 2020s. Legally, back then the new technologies fell into a gray zone. Nevertheless, online surveillance and data collection by the police sparked heated debate not only among legal experts, but also among policymakers and the public. These debates tended to revolve around who was authorized to access data, what data could be collected, and whether the police could collect facial imagery in public spaces. Landmark rulings by the Federal Constitutional Court declared the collection and sharing of data by the police illegal, and ordered such measures to be stopped. The European Directive on the Ethical Use of AI, ratified in 2030, was adopted into German law in 2032, and finally offered clarity. In subsequent years, numerous cases came to light in which the police had inappropriately accessed data. Among other things, security officials had attempted to gain access to the telecommunications data of private companies on the grounds that this was necessary to protect national security. While such activities were deemed permissible by some regional courts, these rulings were ultimately overturned by higher courts again and again.
Individuals have the right to view the data used to calculate their social score. However, hardly anyone does this, as public trust in the system is very high. Furthermore, there are no high profile instances of an individual suffering major disadvantages from his or her social score. Most people are ignorant of the scope and nature of data collection conducted by public authorities. Furthermore, the scoring process is extremely elaborate. Accordingly, the vast majority of individuals fail to see the point in requesting access to the data maintained about their person.	*Social Scoring Systems*	In 2035, there is high public awareness for the right to data privacy, thanks to numerous prominent court rulings. While government data collection has been minimal over the last fifteen years and a government-run social scoring system remains unthinkable, private social scoring systems have been hotly debated since 2032. In 2034, a law was passed to make the data collected by credit-reporting agencies, health insurers, and other companies more transparent. In addition to viewing stored data, individuals can request information about scoring methods, and can also have incorrect data revised or, in some cases, deleted. For example, Irene S. was able to have her health insurance company delete information about her non-participation in a subsidized sports program, because, as a single working mother, she did not have sufficient free time. This deletion reduced her monthly insurance premium.

## Conclusion

Two contrasting sociotechnical imaginaries were elaborated in the foregoing paper. In the first scenario, we considered a security-oriented society that is quick to embrace “technological fixes.” In the second scenario, by way of contrast, we examined a somewhat more traditionalist society that is skeptical of technology and more tolerant of risk.

The dynamic factors that we identified at the outset of the paper are characterized by a certain degree of ambivalence. Specifically, our dynamic factors have the potential to drive future developments, yet are also dependent themselves on specific developments. Our findings indicate that regulatory decisions in combination with international norms and standards will play an important role in shaping future developments with regard to an increasing or decreasing use of security technologies. At the same time, concrete developments “on the ground” that are enabled by technical advancements are likely to be an important influencing factor. To provide one example, the adoption of proactive surveillance technologies by the police, once firmly established, could become difficult to reverse.

In this way, the future that ultimately manifests will be the crucially conditioned by the decisions taken by legislators and the courts. At the same time, the concrete technological possibilities that are realized at any given time will play an important mediating role. These mutually independent key factors will set the stage for future developments. In this way, any effort to bring about a desired set of future relationships in the domain of security must necessarily devoted special attention to the political culture that we collectively foster and the legal regulations that emerge therefrom. Such considerations, in turn, highlight the difficulty at arriving at a firm definition of a “secure future”. Security cannot be assessed on a one-dimensional continuum; we cannot say conclusively that the “To Be Ahead” scenario is the more “secure” one. The definition of “security” is naturally subject to a process of political negotiation, as vividly demonstrated by our contrasting projections of future developments. Our scenarios illustrate the practical implications of security technologies for the populace while also casting light on their intended and unintended consequences.

The presented scenarios are particularly valuable with a view to the divergent alternative futures they present. By definition, both futures cannot occur simultaneously. In this way, they illuminate alternative paths on which we might embark, herein resides their true value. By distilling relevant developmental factors and projecting them forward within the scope of internally coherent, tangible narratives, we provide a foundation for further deliberation. Indeed, for knowledge to be successfully disseminated and invested with practical value, it is necessary to encourage its adoption on the part of addressees. In this context, “knowledge transfer” refers to the communication of research findings to an audience such as policy makers. However, the process of transfer cannot directly be observed, as it tends to take place in an unstructured manner. In addition, the acceptance and dissemination of knowledge is influenced by various factors, and the knowledge transferred is often not applied in a direct manner [14]. Effective science communication informs about the consequences of decisions, including the associated risks, costs, and benefits. Accordingly, it aims to furnish a shared basis of understanding, and thus enable recipients make informed decisions [12]. Scenarios—such as those presented in this paper—are a means of supporting this goal.

In this way, our paper aims to provide a concrete, empirically basis for discussions regarding our future; our scenarios are also illustrative, as they furnish practical points of reference for the consideration of possible policy strategies. By acting as a “policy entrepreneur” and using “policy windows”, one can arrange talks, discussions, and workshops with decision makers. These deliberations should concentrate on the key factors identified in this study—that is, on the factors that are highly relevant in the scenario process. A focus should lie on dynamic key factors and driving factors (Quadrant I and II, Fig. 2), because the key factors have strong reciprocal relationships, and the driving factors in particular operate as a “lever” or catalyst for future developments. Focusing on these issues can help to avoid getting sidetracked in less relevant discussions. It is our aim that the presented scenarios will serve as a springboard for broader public discussion regarding various aspects of security technology, for—as our scenarios indicate—such technologies have the potential to exert major impacts on the domains of politics, society, and the economy.

### Methodical reflections

In developing the scenarios, we sought to ensure that our imagined futures satisfied numerous quality criteria. Specifically, we aimed to ensure they were plausible, consistent, understandable, traceable, distinctive, integrated, triangulated, and theoretically grounded (cf. [13, 16, 30, 31, 36]). We have ensured that our scenarios are internally coherent by conducting impact assessments and associated consistency analysis. Our scenarios represent divergent development paths, which makes them clearly distinctive. Our overall research process was disclosed at the outset and can thus be traced at every step. We, the authors, worked in unison to complete each step. Individual methodological sections were at first undertaken separately, in order to identify similarities and points of contrast. Nevertheless, we allow that an alternatively composed research team could potentially arrive at scenarios with different features. The data compiled in our literature review could have permitted the elaboration of more than two scenarios. However, this would have complicated a clear delineation between the scenarios.

Our scenarios should be understood as a tool for enabling communication. Specifically, the narratives sequences and their content seek to facilitate to “meaning making” [8]. While the scenarios we have described intend to enable negotiative processes, a scenario development approach can never be suitable for arriving at a final or conclusive assessment of a subject matter. In this way, far from seeking to adopt a specific polemic tact, we aim instead with this paper to provide impetus to debates about our collective future.

## Data Availability

The datasets used and analyzed during the current study are available from the corresponding author on reasonable request.

## Footnotes

1 Translated by the authors: “Techniken sind künstlich erzeugte und in der einen oder anderen Weise festgelegte Wirkungszusammenhänge [zu verstehen], die genutzt werden können, um hinreichend zuverlässig und wiederholbar bestimmte erwünschte Effekte hervorzubringen.”

2 Based on our content review, we identified various relevant technological developments, which can be subsumed under the following categories: networked databases; facial recognition; prisoner tracking devices; body cams; video surveillance; predictive policing; mobile-phone location tracking; genetic analysis; online surveillance.

3 Based on our content review, we identified various possible developments: expanded powers for security authorities; new security technology databases or institutional resources; misuse of security technologies; lack of transparency; no popular resistance to expanded use of security technologies; use of surveillance measures without concrete suspicion; facial recognition order by judge; violation of fundamental rights; no data retention; police use of corporate security technologies; corporate control of security technologies used by government authorities.

4 The parameters are from the software manual and were determined on the basis of research and evaluation.

## References

[1] Bartl G (2016). Die subjektive Wahrnehmung und Bewertung von Sicherheitsmaßnahmen an Flughäfen als soziale Reflexion des Verhältnisses zwischen Freiheit und Sicherheit?. Zeitschrift Für Außen- Und Sicherheitspolitik.

[2] Bauman Z (2007). Liquid times: living in an age of uncertainty.

[3] Beck U (1986). Risikogesellschaft: auf dem Weg in eine andere Moderne.

[4] Buzan B, Wæver O, de Wilde J (1998). Security: a new framework for analysis.

[5] Ceyhan A (2008) Technologization of security: management of uncertainty and risk in the age of biometrics. Surveillance & Society 5(2). 10.24908/ss.v5i2.3430.

[6] Collingridge D (1980). The social control of technology.

[7] Daase C (2010) Wandel der Sicherheitskultur. Aus Politik und Zeitgeschichte: Sicherheitspolitik 50:9–16

[8] Davies SR, Halpern M, Horst M, Kirby DA, Lewenstein B (2019) Science stories as culture: experience, identity, narrative and emotion in public communication of science. J Sci Commun 18(5). 10.22323/2.18050201

[9] Egbert S (2018). Predictive Policing in Deutschland. Grundlagen, Risiken, (mögliche) Zukunft. Strafverteidigervereinigungen (Chair), Texte und Ergebnisse des 42.

[10] Egbert S (2018). About discursive storylines and techno-fixes: the political framing of the implementation of predictive policing in Germany. Eur J Secur Res.

[11] Fink A, Marr B, Siebe A, Kuhle J-P (2005). The future scorecard: combining external and internal scenarios to create strategic foresight. Manag Decis.

[12] Fischhoff B (2013). The sciences of science communication. Proc Natl Acad Sci U S A.

[13] Flick U (2019). An introduction to qualitative research (6th ed).

[14] Froese A, Simon D, Froese A, Simon D, Böttcher J (2016). Eine disziplinäre Perspektive auf Wissenstransfer - zu Einführung. Science studies. Sozialwissenschaften und Gesellschaft: Neue Verortungen von Wissenstransfer. Transcript, Bielefeld.

[15] Gausemeier J, Fink A, Schlake O (1996). Szenario-management: Planen und Fuehren mit Szenarien (2nd ed. revisited).

[16] Gerhold L, Holtmannspötter D, Neuhaus C, Schüll E, Schulz-Montag B, Steinmüller K, Zweck A (2015). Standards und Gütekriterien der Zukunftsforschung.

[17] Gerhold L, Peperhove R, Brandes E, Hughes AL, McNeill F, Zobel C (2020). Using scenarios in a living lab for improving emergency preparedness. WiP paper – planning, foresight and risk analysis.

[18] Gerhold L, Steinmüller K, Peperhove R, Steinmüller K, Dienel L (2018). Security 2025: scenarios as an instrument for dialogue. Envisioning uncertain futures (Zukunft und Forschung 6).

[19] Gillespie T, Gillespie T, Boczkowski PJ, Foot KA (2014). The relevance of algorithms. Media technologies: essays on communication, materiality, and society.

[20] Gramigna R, Marling R (2018). Scenario as a tool for critical thinking: climate change awareness and denial as a case study. ESSACHESS- J Commun Stud.

[21] Grishakova M, Gramigna R, Sorokin S (2019). Imaginary scenarios: on the use and misuse of fiction. Front Narrat Stud.

[22] Häußling R, Kneer G, Schroer M (2010). Techniksoziologie. Handbuch Spezielle Soziologien.

[23] Hussain M, Tapinos E, Knight L (2017). Scenario-driven roadmapping for technology foresight. Technol Forecas Soc Change.

[24] Introna LD, Wood DM (2004). Picturing algorithmic surveillance: the politics of facial recognition systems. Surveill Soc.

[25] Jasanoff S, Kim S-H (2009). Containing the atom: sociotechnical imaginaries and nuclear power in the United States and South Korea. Minerva.

[26] Jasanoff S, Kim S-H (2015). Dreamscapes of modernity: sociotechnical imaginaries and the fabrication of power.

[27] Kasperson RE, Renn O, Slovic P, Brown HS, Emel J, Goble R, Ratick S (1988). The social amplification of risk: a conceptual framework. Risk Anal.

[28] Kaufmann S (2016). Security through technology? Logic, ambivalence and paradoxes of technologised security. Eur J Secur Res.

[29] Kaufmann S, Gusy C, Kugelmann D, Würtenberger T (2017). Das Themenfeld “Zivile Sicherheit”. Rechtshandbuch Zivile Sicherheit.

[30] Kosow H (2015). New outlooks in traceability and consistency of integrated scenarios. Eur J Futures Res.

[31] Kosow H, Gaßner R (2008). Methods of future scenario analysis. Overview, assessment and selection criteria. DIE studies 39.

[32] Latour B (2016). Der Berliner Schlüssel (3. Auflage). Locked: no. 1.

[33] Lutz B, Lutz B (1987). Das Ende des Technikdeterminismus und die Folgen: soziologische Technikforschung vor neuen Aufgaben und neuen Problemen. Technik und sozialer Wandel: Verhandlungen des 23. Deutschen Soziologentages in Hamburg 1986.

[34] Marx G. T (2002) What’s new about the “new surveillance”? Classifying for change and continuity. Surveill Soc 1(1): 9–29. 10.24908/ss.v1i1.3391

[35] Matsuzaki H, Lüdtke N, Matsuzaki H (2011). Die Frage nach der “agency” von Technik und die Normenvergessenheit der Techniksoziologie. Akteur – Individuum – Subjekt.

[36] Mayring P (2016). Einführung in die qualitative Sozialforschung. Eine Anleitung zum Denken.

[37] Menno Harms J, Zoche P, Kaufmann S, Haverkamp R (2010). 3. Sicherheitsgewinn mit technologischen Innovationen (Schwerpunkt ITK). Zivile Sicherheit.

[38] Meyer C (2017) Digitale Disziplin. PROKLA. Zeitschrift Für Kritische Sozialwissenschaft 47(186). 10.32387/prokla.v47i186.179

[39] Mittelstadt BD, Allo P, Taddeo M, Wachter S, Floridi L (2016). The ethics of algorithms: mapping the debate. Big Data Soc.

[40] Neuhaus C, Gerhold L (2015). Prinzip Zukunftsbild. Standards und Gütekriterien der Zukunftsforschung.

[41] Orwat C (2019). Diskriminierungsrisiken durch Verwendung von Algorithmen: Eine Studie erstellt mit einer Zuwendung der Antidiskriminierungsstelle des Bundes (1. Auflage).

[42] Pilkahn U, Aktiengesellschaft S (2008). Using trends and scenarios as tools for strategy development. Shaping the future of your enterprise.

[43] Rammert W (1993). Technik aus soziologischer Perspektive.

[44] Rammert W, Maurer A (2008). Technik und innovation. Wirtschaft + Gesellschaft. Handbuch der Wirtschaftssoziologie.

[45] Rammert W (2016). Technik - Handeln - Wissen: Zu einer pragmatistischen Technik- und Sozialtheorie (2. Aufl. 2016).

[46] Rammert W, Rammert W (2016). Technik, Handeln und Sozialstruktur. Technik - Handeln - Wissen.

[47] Renn O, Burns WJ, Kasperson JX, Kasperson RE, Slovic P (1992). The social amplification of risk: theoretical foundations and empirical applications. J Soc Issues.

[48] Rohberge J, Seyfert R (2017). Algorithmuskulturen. Über die rechnerische Konstruktion der Wirklichkeit.

[49] Schulz-Schaeffer I (1999). Technik und die Dualität von Ressourcen und Routinen: zur sozialen Bedeutung gegenständlicher Technik. Z Soziol.

[50] Schulz-Schaeffer I, Baur N, Korte H, Löw M, Schroer M (2008). Technik. Handbuch Soziologie.

[51] Schulz-Schaeffer I, Rammert W, Schubert C, Schulz-Schaeffer I (2019). Technik, Handeln und praxis. Das Konzept gradualisierten Handelns revisited. Berliner Schlüssel zur Techniksoziologie.

[52] Steinmüller K (2012). Szenarien – Ein Methodenkomplex zwischen wissenschaftlichem Anspruch und zeitgeistiger Bricolage in Zukunft und Wissenschaft. Popp R (ed), Zukunft und Wissenschaft.

[53] Stewart J, Williams R (1998). The coevolution of society and multimedia technology. Soc Sci Comput Rev.

[54] Suchman L, Follis K, Weber J (2017). Tracking and targeting. Sci Technol Hum Values.

[55] von Reibnitz U (1992). Szenario-Technik. Instrumente für die unternehmerische und persönliche Erfolgsplanung.

[56] Washida Y, Yahata A (2021). Predictive value of horizon scanning for future scenarios. Foresight.

[57] Würtenberger T, Tanneberger S, Winzer P, Schnieder E, Bach F-W (2010). Gesellschaftllche Voraussetzungen und Folgen der Technisierung von Sicherheit. Sicherheitsforschung-Chancen und Perspektiven.

[58] Zurawski N (2015). Technische Innovationen und deren gesellschaftliche Auswirkungen im Kontext von Überwachung. Schriftenreihe Sicherheit (Vol. 16).

